# Impact Model-Based Physical-Activity Promotion at the Workplace: Study Protocol for a Mixed-Methods Study in Germany (KomRueBer Study)

**DOI:** 10.3390/ijerph18116074

**Published:** 2021-06-04

**Authors:** Andrea Schaller, Carina Hoffmann

**Affiliations:** 1 Working Group Physical Activity-Related Prevention Research, Institute of Movement Therapy and Movement-Oriented Prevention and Rehabilitation, German Sport University Cologne, Am Sportpark Muengersdorf 6, 50933 Cologne, Germany; a.schaller@dshs-koeln.de; 2 Department Research and Development, Institute for Occupational Health Promotion, Neumarkt 35-37, 50667 Cologne, Germany

**Keywords:** workplace, health promotion, physical activity, impact model, mixed methods, complex interventions

## Abstract

There is great potential for the implementation of physical-activity measures at the workplace, especially in smaller companies. The present paper describes the study protocol for evaluating an impact-model-based multicomponent intervention promoting physical activity at the workplace within a cross-company network. The evaluation is based on a logic model focusing on outputs and short-term outcomes for the purpose of physical-activity promotion, physical-activity-related health competence, and knowledge about physical activity. A mixed-methods approach is applied. The quantitative evaluation is conducted as a natural design, and combines a retrospective evaluation of the acceptance, usage, and satisfaction (output) at the end of the measures, and two surveys that capture physical activity, knowledge about physical activity, and physical-activity-related health competence (outcome) of the employees in the form of a trend study. The qualitative evaluation comprises semistructured interviews to investigate knowledge of the existence of and attitude towards the content of the multicomponent intervention and the study. The challenges evaluating complex interventions are widely debated. Through an impact-model-based approach, the study will provide a promising framework for the systematic evaluation of a multicomponent intervention promoting physical activity.

## 1. Introduction

The individual health consequences of physical inactivity [1,2,3,4,5,6] and the related socioeconomic burden [7,8,9] are well-known. Consequently, the importance of exercise and physical-activity promotion [10,11] is widely acknowledged.

A recognised setting for health promotion is the workplace [12,13]. In Germany, the Prevention Act [14], which came into force in July 2015, underlines the importance of workplace health promotion (WHP). This can be seen, for example, in the expenditure of statutory health-insurance funds. Expenditures on WHP rose from EUR 68 million in 2014 [15] to over EUR 230 million in 2019 [16], and the number of reached companies increased by 104% to 23,221 [15,16]. The content of the interventions within WHP could thereby focus on environmental and behavioural measures [17]. Environmental measures include the categories of the health-promoting design of work activities and conditions, the health-promoting design of operational conditions, and healthy leadership, and consider the design of workers’ conditions [17]. Behavioural measures, on the other hand, address individual coping skills and encompass the categories of physical-activity-promoting work and physically active employees, stress-management and -strengthening resources, healthy diet in everyday work, and addiction prevention [17]. In the field of WHP, 45% of environmental measures and 69% of behavioural measures could be assigned to the field of physical activity in 2019 [16]. In particular, multicomponent approaches that combine environmental measures on the structural and process levels (e.g., the movement-friendly redesign of work processes, and the creation of infrastructures that promote physical activity), and behavioural measures (e.g., courses and exercise programmes) are considered to be promising [4]. Even though the workplace is regarded as a promising approach to promote physical activity among adults [4,18], studies on sustainable and effective interventions are still considered to be limited, and most reviews show inconclusive results [19,20,21,22]. The methodological quality of studies is also often limited [21,22].

Basically, WHP implementation in small and micro companies remains a big challenge [23,24,25,26]. WHP financed by statutory health-insurance funds, is most frequently implemented in companies with 100 to 249 employees [16]. Cross-company networks are considered to be a promising approach to counteract this problem [16]. This approach was introduced in 2014 in the guideline on prevention as a field of action called “cross-company networking and consultation” with the prevention principle “dissemination and implementation of workplace health promotion through cross-company networks”, and was again significantly strengthened by the Prevention Act [27]. In this way, knowledge and resources are pooled at the network level, so that small and micro companies in particular can benefit from WHP [28]. Within the promotion of physical activity, the network concept is also of great interest in health policy, and is systematically processed on the national level [29].

The KomRueBer study is part of funding priority Exercise and the Promotion of Physical Activity by the Federal Ministry of Health (BMG), which aims to make the health-benefit potential of physical activity known to the entire population via different settings [30]. In this context, the KomRueBer study focuses on the workplace and especially the promotion of physical activity for employees in smaller companies [30]. For this purpose, the study takes advantage of the promising approach of cross-company networks as described above. Thus, the overall objective of the KomRueBer study is the conception, implementation, and evaluation of a theory-based cross-company network for promoting physical activity [31]. The KomRueBer study is composed of two parts. In the first part (conception phase), the cross-company network and multicomponent intervention for promoting physical activity were developed over the course of 9 months (July 2019 to March 2020) [31]. In order to identify the requests and requirements of various stakeholders with regard to the cross-company network and the planned multicomponent intervention for promoting physical activity, a mixed-methods approach was applied [31]. There is a separate ethical approval for the conception phase, and the results and applied measures were published [31]. In the second part of the KomRueBer study, the multicomponent intervention is implemented and evaluated (implementation phase).

The present study protocol describes the framework and methods of the evaluation within the implementation phase, whereby the evaluation of measures promoting physical activity at the individual level is focused. Measures on the organisational level within the KomRueBer study (e.g., network activities; see [31]) are separately evaluated within further research and by means of social-network analysis [32].

The main questions are: (1) How is the acceptance and usage of the measures promoting physical activity? (2) What are the facilitating factors to and barriers from participation in the measures from the employees’ point of view? (3) How do the employees perceive the KomRueBer project and the associated physical-activity measures?

## 2. Materials and Methods

The KomRueBer study aims at promoting physical activity during the daily working routine and in leisure time, enhancing physical-activity-related health competence, and increasing knowledge about physical activity. The study is conducted in compliance with the Helsinki Declaration and was approved by the Ethics Committee of the German Sport University Cologne (reference number 068/2020). It is registered in the German Clinical Trials Register (DRKS00020956). The recruitment of participants started in August 2020 and will be completed in May 2022. Informed consent is taken from each participant.

### 2.1. Conceptual Framework

**Figure 1:**
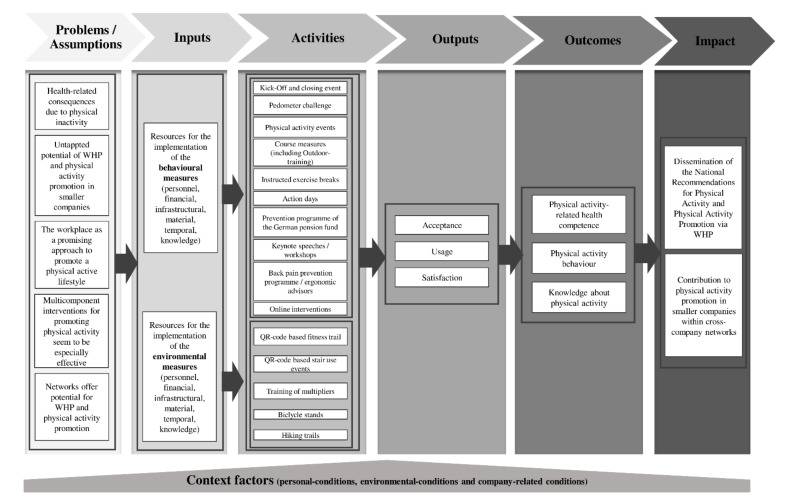
Logic model of KomRueBer study (individual level).

The evaluation of the implementation phase is based on a logic model that allows for the systematic examination of the relationship between concept planning and impact [33,34]. The core element of the logic model is the assumption of a cause–effect chain. It is based on theoretical assumptions regarding interventions (Assumptions; Theory) and shows with which means and resources (Input) which measures (Activities) are carried out. It also shows, with regard to the measures, how they are used (Output) and what effects are achieved with the target group (Outcome) on the meso–macro level (Impact) [33]. Context factors in terms of personal, environmental, and company-related conditions are also considered to be possible influencing factors. Figure 1 shows the *assumptions* (see Problems/Assumptions) and planned *impact* behind the KomRueBer multicomponent intervention. As a project of funding priority Exercise and the Promotion of Physical Activity, the superior aim (*Impact)* of the KomRueBer study is a contribution to the dissemination of the national recommendations for physical activity and physical activity promotion [4,30]. Thereby, the project focuses on the dissemination in the workplace setting. Furthermore, the logic model shows the *inputs*, including available resources for the various measures to promote physical activity, the undertaken *activities*, the *outputs* comprising the acceptance, usage, and satisfaction of the employees, and lastly, the target *outcomes* as a result of the multicomponent intervention. The offered *activities*, respectively measures were developed to be participative in the conception phase [31].

The logic-model-based evaluation focuses on programme activities, outputs, and short-term outcomes for the purpose of physical-activity promotion, physical-activity-related health competence, and knowledge about physical activity. Therefore, a mixed-methods approach comprising quantitative and qualitative methods is applied.

### 2.2. KomRueBer Multicomponent Intervention (Activities)

The intervention was developed in a participatory manner and was described by Hoffmann et al. [31]. As development was finished in February 2020 before the COVID-19 pandemic affected life in Germany, additional online interventions were included. All measures are carried out by local exercise and WHP providers, and in consensus with the Prevention Guidelines of the Central Federal Association of the Health Insurance Funds [17,35]. Due to the prolonged pandemic, it is unclear when on-site measures can actually begin.

**Figure 2:**
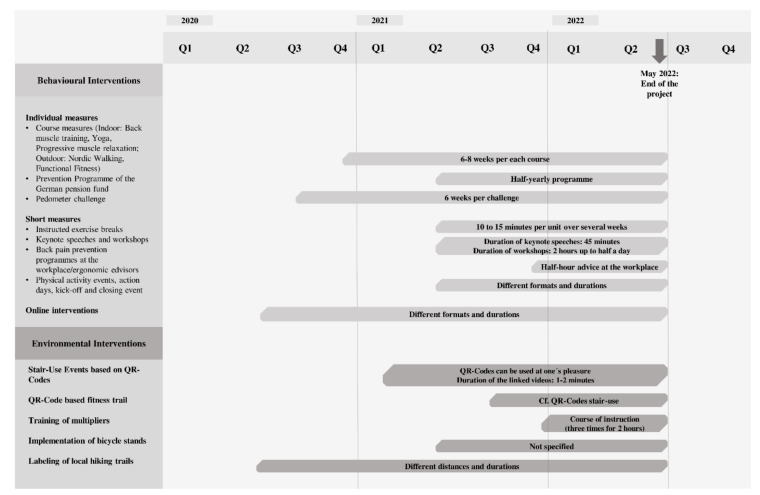
Measures and timetable of measures to promote physical activity in KomRueBer. Note: Q, quarter.

Within behavioural measures, a distinction is made between individual measures (long-term measures; lasting 6–8 weeks or longer, e.g., courses) and short measures (one-off measures of short duration or intermittent participation that take place on site, e.g., workshops, instructed exercise breaks) (see Figure 2). Next to individual and short measures, online interventions are the third component within behavioural measures. On the one hand, they include short measures that can be digitally implemented (e.g., lectures) and where registration via website is necessary. On the other hand, they include measures that can be digitally implemented, but where a registration is not necessary (e.g., reference to measures of third parties or digital-information supply such as the description of regional sports clubs). Courses that can be offered online during the COVID-19 pandemic are counted among individual measures. Further information on the duration of respective measures can be found within the timeline. Most interventions are offered repeatedly over the entire project period. However, there are regular interruptions in order to be able to react to determining factors (e.g., changed needs, financing). The measures are advertised via newsletters, e-mails, posters, and a project website.

### 2.3. Participants and Setting

The target group of the multicomponent intervention comprises around 2000 employees from different companies in a technology park in Germany, and was described by Hoffmann et al. [31]. Participation in the physical-activity measures and the evaluation are voluntary and can be revoked at any time. Nonparticipation in the interventions and/or the accompanying evaluation has no negative consequences for the participants. Participants are informed in writing and/or orally about the accompanying evaluation at the beginning of the measure. Within the context of online evaluations, participation in the evaluation is preceded by consent of the described conditions (via checkbox).

The inclusion criteria for participation in all physical-activity programmes are: (1) an employment relationship in a company of the technology park, (2) no sick leave, (3) age 18 to 67 years, and (4) written informed consent to participate in the study. “No sick leave” is independently assessed with regard to the continued payment of wages. Regardless of duration, it refers to the fact that someone who is on sick leave, is also unable to participate in WHP measures.

Exclusion criteria are defined in line with the Prevention Guidelines of the Central Federal Association of the Health Insurance Funds [17,35] (prevention principle “reduction of lack of physical activity through health-related sports activity”) and comprise (1) diseases of the musculoskeletal system requiring treatment. Beyond, the following exclusion criteria were defined: (2) the indication for rehabilitative treatment, (3) the need for acute care, and a (4) lack of understanding of the German language. The inclusion of the first study participant was on 21 August 2020. The recruitment period is planned until May 2022.

### 2.4. Data Collection

#### 2.4.1. Quantitative Evaluation

The quantitative evaluation is conducted as a natural design trial and focuses on the output level (see impact model, Figure 1) to capture the acceptance, usage, and satisfaction with the respective measure. Table 1 shows the measure-specific outputs for gathering the usage and corresponding operationalisation of the behavioural measures. The outputs and operationalisation of environmental measures are shown in Table 2. Basically, the measures are seen in relation to the time. Participants’ acceptance of and satisfaction with the measure are formatively evaluated by means of different surveys.

Behavioural measures lasting several weeks (see Table 1, individual measures) and short measures (see Table 1) are evaluated at the end of the respective measure. The evaluation is anonymously offered in pencil-and-paper form or as an anonymous online survey, depending on the situation of the pandemic. The aim of the surveys is to capture acceptance and satisfaction with the respective measure (output). Within the evaluation of individual measures, physical-activity-related health competence is likewise collected. Online and environmental interventions are evaluated according to the outputs listed in Table 1 and Table 2.

To ascertain the acceptance, usage, and satisfaction (output) of the entire multicomponent intervention, two anonymous online employee surveys are planned; one was conducted in April 2021 (interim survey) and the other will be conducted in March 2022 (final survey). The surveys assess physical activity, knowledge of physical activity, and the physical-activity-related health competence of employees (outcome) in the form of a trend study. The surveys are addressed to all employees on site.

On the basis of the results of the participatory conception phase [31], a sample of around 250 employees is expected at each time of measurement (April 2021, March 2022). The number of participants within the evaluation of individual and short measures depends on the number of employees participating in the respective measures.

The used instruments in the different surveys are listed in Table 3. Participation in all evaluations is on a voluntary basis and can be cancelled or revoked at any time. For all online surveys, online survey tool EFS Survey (Questback GmbH), which is well-established in the academic field, is used. All data are anonymously collected.

##### Data Analysis

Descriptive statistics are conducted to describe the characteristics of a study population and explore variable distributions on the individual level.

Each measure (activity) is independently evaluated. Therefore, descriptive statistics (means (mean), standard deviations (±SD), frequencies (*n*) and percentages (%)) are used to characterise and describe the results on the output level. Second, gender-specific differences in the output variables of each measure are examined if available. Depending on the data distribution, parametric or nonparametric statistical tests are used to evaluate group differences in output variables.

The overall multicomponent intervention is evaluated by cross-sectional survey. The results of the outcomes are presented as means (mean) and standard deviation (±SD) for continuous data, and as frequency tables (*n*; %) for categorical data. Multiple-regression analyses are conducted to identify associations of possible factors (sociodemographic factors, usage of activities) influencing the outcomes of physical activity, movement-related health competence, and knowledge about physical activity.

#### 2.4.2. Qualitative Evaluation

The qualitative evaluation comprises semistructured interviews with employees on site. Objectives of the qualitative evaluation include investigating and determining knowledge of the existence and content of the multicomponent intervention and the KomRueBer study. Therefore, the qualitative evaluation contributes to research questions (2) and (3).

The semistructured interviews are conducted to obtain a deeper insight into the perspective of the employees on the KomRueBer project and related physical-activity measures. Additionally, the interview guideline addresses facilitators and barriers to the usage of the measures. By the use of open-ended questions, we expect to gain more detailed and enriched qualitative data on the usage and acceptance of the multicomponent intervention, including both barriers and enablers to physical activity. The interviews are conducted in German and lead by one researcher. They are anonymised by using a code and audiorecorded. Participation in the interviews is on a voluntary basis and can be cancelled or revoked at any time.

An exact sample size for the qualitative evaluation can only be ascertained during the project progress and it is based on theoretical saturation. A minimal sample of 15 participants is required. The interviews are transcribed according to Dresing and Pehl [44], double-checked, and evaluated by means of structuring content analysis [45,46]. Evaluation is carried out with MAXQDA 11 software (VERBI GmbH, Berlin, Germany).

##### Triangulation of Quantitative and Qualitative Data

**Figure 3:**
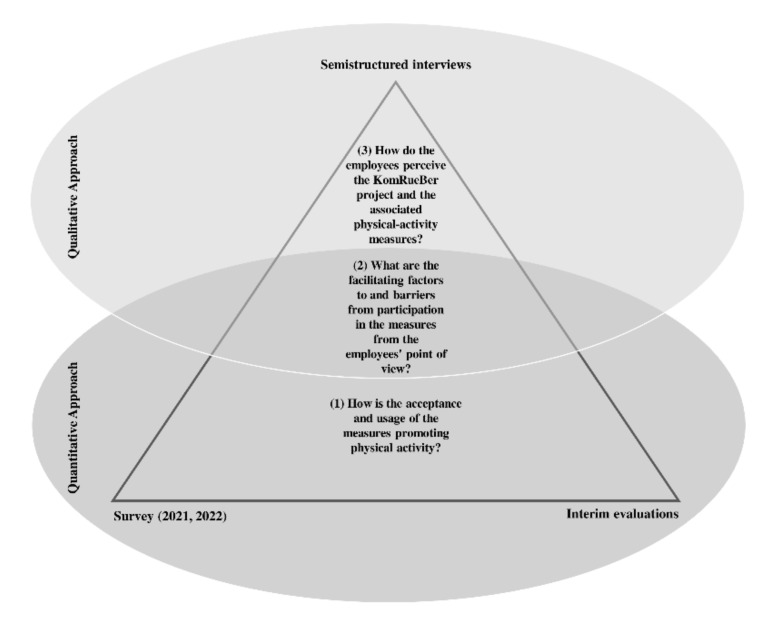
Triangulation of qualitative and quantitative data within KomRueBer study.

Different types of data (numerical, text) are generated from different methodological approaches (qualitative, quantitative). For a well-founded answer to the research questions, triangulation of the qualitative and quantitative data is carried out (see Figure 3). Triangulation enables us to develop an overall interpretation of the implementation stage of the KomRueBer study. Qualitative and quantitative data complement each other, thereby giving insight into the course of KomRueBer. The survey (April 2021), interim evaluations of individual and short measures, and the online and environmental interventions enable capturing the outputs. The results of this quantitative approach contribute to the development of the interview guideline. The interviews themselves then contribute to a deeper insight of the survey results. The methodological approaches thus both contribute to answering the research questions independently of one another, and enable a comprehensive understanding of physical-activity promotion in cross-company networks and its impact on the individual outcome level.

## 3. Discussion

The aim of the study is the impact-model-based evaluation of a multicomponent intervention promoting physical activity at the workplace. On the practical level, the study will provide important information on the usage and acceptance with the applied measures, and identifiy facilitators and barriers to the utilisation of physical-activity measures by employees within WHP in a cross-company network.

Overall, the importance of prevention and health promotion is undisputed [13]. Therefore, a complex system of prevention and health promotion has emerged in Germany, in recent years [47]. As a consequence, expectations on this sector have risen [48], and the demand for evidence is central [47]. However, to exploit the full potential of this sector, De Bock et al. [47] emphasise that challenges such as the mere temporary and local development of measures, the untapped potential of evaluating measures, and the unsystematic use of scientific knowledge have to be resolved. Overall, there is a great demand for better evaluation and reporting in the context of public health, also comprising physical-activity promotion [48,49,50,51]. Despite promising indications on the effectiveness of physical-activity measures at the workplace [4,20], the evaluation of public-health interventions still implicates challenges, especially for practitioners [52].

Changes in behaviour require measures at various levels [53]; thus, public-health measures are often complex [47,51]. As Kolip [54] states, physical-activity interventions can also be considered to be complex interventions. Complex interventions comprise several components [55,56,57], could vary in terms of their outcomes [57], and are mostly applicable in complex contexts [55]. Rütten et al. [58] describe the promotion of active lifestyles as a coproduction of various stakeholders. This once again emphasises the complexity that is at the basis of this kind of intervention. The literature [48] refers to the importance of also taking this complexity into account within the evaluation, but it is undisputed that the evaluation and the attribution of the impact of complex interventions poses challenges [51,55,56,57,59]. Frequently, the application of RCTs is part of the discourse [48].

As previous research indicates, evaluation frameworks can help to cope with these challenges, and support the design and evaluation of complex interventions [47,56,60,61,62,63]. Logic models such as the one presented in the KomRueBer study are also useful in both programme planning and evaluation [33,64,65,66]. Within the evaluation of a programme, logic models can help to accomplish a basic understanding of the respective programme [65] and in the further course support focusing on meaningful evaluation questions and their formulation from different angles [33]. A logic model makes it easier to coordinate the evaluation instruments with the programme goals, and this allows for the more precise recording of the extent to which goals are achieved [67]. Thus, logic models can help to design an evaluation plan that focuses the evaluation on the most important dimensions of the programme [68], and consequently allows for the improvement of data collection [66]. To conclude, according to Reynolds and Sutherland [69], logic models can contribute to an evidence base for what works within programmes.

There is a large amount of research that the usage of impact models in the entire process, from planning to implementation and evaluation, provides benefits. However, some limitations have to be stated. As Knowlton and Phillips [70] note, it must not be assumed that the respective model really produces the desired results and is ready for implementation. Rather, they should be used critically and questioning [63]. Balthasar and Fässler [71] identified existing limits, including the fact that impact models can lead to simplification and hence do no justice to complex interventions. They also argued that the influence of the context factors on the impact of a programme may not be sufficiently taken into account [71]. Understanding the context factors of a programme is a particular focus of the theory of change [72]. It is frequently used to plan, implement, and evaluate various programmes and project strategies [73], particularly in international development, for larger initiatives and for more complex programmes [72]. Similar to the logic model, the theory of change is based on representing the manner of functioning of a programme [74]. While logic models can inveigle to simplification [71], the theory of change is far more detailed and highlights the assumptions behind each step [72,74]. However, the use of a logic model for the present study appears appropriate, as the focus is on the programme itself. Nevertheless, it is important for this study to consider the context factors because they can help to explain the weaknesses, strengths, and possible influences of the programme [33,66].

In summary, the KomRueBer study will address challenges concerning complex interventions and their evaluation outlined above. Like the majority of interventions in WHP, the KomRueBer multicomponent intervention can also be rated as a complex intervention. It consists of different components and can basically vary in terms of their outcomes; it is implemented in a complex setting with a variety of stakeholders. The KomRueBer study will address this complexity through its model-based evaluation and presentation of results within the scope of a logic model. Consequently, the study will provide a descriptive roadmap that shows which activities lead to which outputs and which measures are well-accepted by the target group (e.g., differentiated according to topics and formats). This will enable empirical statements about the usage and acceptance of various measures to promote physical activity within WHP, thus creating a basis for discussion for all stakeholders. In order to determine how people can be motivated for physical activity, this practical implementation study provides a valuable empirical contribution, alsofor the transfer of such model projects to other regions. Thus, this study protocol is the interface between the participatory development of WHP measures and a later participatory interpretation of results with the stakeholders of the cross-company network.

### Risk Factors and Limitations

There are some challenges in conducting this study. A low level of willingness to participate in WHP interventions is a well-known challenge [75]. Although in the conception phase [31] value was made on the participatory development of the multicomponent intervention, a low participation rate cannot be excluded. Depending on the actual pandemic situation, the availability and direct addressing of employees on site is only possible on a limited scale. Different personnel-related, environmental, and company-related conditions (see Figure 1) can influence the study, especially because it is an intervention in a real-life setting. Among others, the functioning of a cross-company network depends on the commitment and motivation of the actors in the network [28]. Thus, participation rates can also depend on the willingness of operational actors to pass on information. Due to the COVID-19 pandemic, online interventions had to be integrated in the multicomponent intervention, even if they were not explicitly requested by the employees. Lastly, recruitment bias, which is a common problem in prevention and health promotion, may also pertain to the present study. There is a risk that only those who had participated in the measures would take part in the surveys. In order to be able to make statements in this regard, questions about previous participation in measures are asked in the surveys. Furthermore, we aim to use the qualitative approach to increasingly reach those employees who did not participate in measures.

Although the real setting implicates a number of challenges, the study adds value to the promotion of physical activity and WHP in smaller companies. Particularly, the triangulation of qualitative and quantitative data is a great strength of the study. It enables a broad picture of possible barriers and facilitators for participation in physical-activity measures at the workplace. As previous research indicates, a broad repertory of methods has proven to be an appropriate and expedient approach, especially within the context of complex interventions [54,76].

## 4. Conclusions

As a model project with a focus on practical implementation research, the study will significantly contribute to the transfer between science and practice, and the advancement of WHP services in the context of physical activity. For implementation research, it will offer a system for improving sustainability and transfer in physical-activity promotion through the impact-model-based presentation of results.

The evaluation approach based on the logic model [33] is used to address the complexity of the described multicomponent intervention, and allows for practitioners and researchers to see what works within this intervention and why. With regard to this interface, such model-based studies will help to more practically illustrate the relationship between design and impact of interventions, and explain to relevant stakeholders how a complex intervention programme works.

## Figures and Tables

**Table 1:** Output and operationalisation of specific behavioural measures of the KomRueBer study.

Activity	Output	Operationalisation
Individual Measures		
Pedometer challenge	Number of pedometer challenges Number of teams per challenge Number of participants per challenge Number of participating companies per challenge Number of finishers per challenge	Registration and list of results
Course measures	Number of courses (subdivided into the different topics) Number of participants per course Number of finishers per course Number of participating companies per course	Registration and documentation sheet
Prevention Programme of the German Pension Fund	Number of lectures to inform about the programme over the entire project period Number of participants in the programme Number of finishers of the programme Number of participating companies	Registration and documentation sheet
Short Measures		
Keynote speeches and workshops	Number of speeches and workshops (subdivided into the different topics) Number of participants per speech or workshop Number of participating companies per speech or workshop	Registration and documentation sheet
Instructed exercise breaks	Number of instructed exercise breaks Number of participants per exercise break	Documentation sheet
Back pain prevention programmes or ergonomic advisors	Number of consultations Number of participants Number of participating companies	Registration and documentation sheet
Physical activity events, action days, kick-off and closing events	Number of measures Number of participants per measure	Documentation sheet
Online Interventions		
Online interventions without registration	Number of online interventions Number of users to the respective online intervention site Number of sessions to the respective online intervention site Number of page impressions to the respective online intervention site	Website analytics
Online interventions with registration	Number of online interventions Number of participants per online intervention Number of participating companies per online intervention Number of users to the respective online intervention site Number of sessions to the respective online intervention site Number of page impressions to the respective online intervention site	Registration and/or website analytics

**Table 2:** Output and operationalisation of specific environmental measures of the KomRueBer study.

Activity	Output	Operationalisation
Stair-use events based on QR codes	Number of events in the context of stair use Number of implemented QR codes in stairwells (indoors) Number of QR code logins (overall and subdivided into the different levels of difficulty or topics)	Documentation sheet and video platform
QR code-based fitness trail	Number of implemented QR codes (outdoors) Number of different fitness trail stations Number of QR code logins (overall and subdivided into the different levels of difficulty or topics)	Documentation sheet and video platform
Training of multipliers	Number of training sessions Number of trained multipliers Number of participating companies	Registration and documentation sheet
Implementation of bicycle stands	Number of new bicycle stands	Documentation sheet
Labelling of local hiking trails	Number of elaborated trails Number of downloaded maps or implemented signs	Documentation sheet and website analytics

**Table 3:** Instruments of quantitative evaluation (individual and short measures, and online surveys).

**Instrument**	**Evaluation Criteria**
Individual Measures	
Questionnaire of physical-activity-related health competence [36]	Physical-activity-related health competence
Finishing questionnaire for health courses of the Central Federal Association of the Health Insurance Funds [37]	Assessment and evaluation of structural quality
Minimum European Health Module (MEHM) [38]	Subjective state of health
German Health Interview and Examination Survey for Adults (DEGS) [39]	Subjective physical activity
Unstandardised questionnaire (further project-related questions)	Acceptance, satisfaction, usage
Unstandardised questionnaire	Sociodemographic and personal variables: sex, age, size of the company
Demographic standards [40]	Employment
German validated version of the European Health Interview Survey-Physical Activity Questionnaires (EHIS-PAQ) [41]	Work activity
Short Measures	
Finishing questionnaire for health courses of the Central Federal Association of the Health Insurance Funds [37]	Assessment and evaluation of structural quality
Unstandardised questionnaire	Sociodemographic and personal variables: sex, age, size of the company
Demographic standards [40]	Employment
German validated version of the European Health Interview Survey-Physical Activity Questionnaires (EHIS-PAQ) [41]	Work activity
Unstandardised questionnaire (further project-related questions)	Acceptance, satisfaction, usage
Survey	
Unstandardised questionnaire	Acceptance, usage, satisfaction, motives and barriers for participation
Questionnaire on the perception of Website content (WWI) [42]	Subjective content perception (pleasure, intelligibility, quality, and utility)
Perceived Website Usability–German (PWU-G) [43]	Subjective usability, user satisfaction
German validated version of the European Health Interview Survey—Physical Activity Questionnaires (EHIS-PAQ) [41]	Muscle strengthening, work activity
German Health Interview and Examination Survey for Adults (DEGS) [39]	Subjective physical activity
Questionnaire of physical-activity-related health competence [36]	Physical-activity-related health competence
Unstandardised questionnaire	Knowledge of physical activity
Unstandardised questionnaire	Sociodemographic characteristics: age, sex, occupational position, height, weight
Demographic standards [40]	Sociodemographic characteristics: employment, educational level
Unstandardised questionnaire	Personal characteristics: type of shift work, size of the company

## Data Availability

No new data were created or analyzed in this study. Data sharing is not applicable to this article.

## German Clinical Trials Register (DRKS)-ID

DRKS00020956; date of registration: 18 June 2020.
